# Ferroptosis – a potential feature underlying neratinib-induced colonic epithelial injury

**DOI:** 10.1007/s00280-024-04699-9

**Published:** 2024-07-13

**Authors:** Triet P. M. Nguyen, Susan L. Woods, Kate R. Secombe, Simon Tang, Aurelia S. Elz, Scott Ayton, John Finnie, Aadya Nagpal, Normand Pouliot, Joanne M. Bowen

**Affiliations:** 1 Present Address: School of Biomedicine, The University of Adelaide, Adelaide, South Australia Australia; 2 Present Address: The Florey Institute of Neuroscience and Mental Health, Melbourne, VIC Australia; 3 Adelaide Medical School, The University of Adelaide, Adelaide, South Australia Australia; 4 School of Life and Environmental Sciences, The University of Sydney, Sydney, Australia; 5 Clinical and Health Sciences, University of South Australia, Adelaide, Australia; 6 Precision Cancer Medicine, South Australian Health and Medical Research Institute, Adelaide, SA Australia; 7 Discipline of Anatomy and Pathology, Adelaide Medical School, University of Adelaide, Adelaide, South Australia Australia; 8 Olivia Newton-John Cancer Research Institute, Heidelberg, VIC Australia; 9 School of Cancer Medicine, La Trobe University, Bundoora, VIC Australia; 10 Department of Clinical Pathology, The University of Melbourne, Melbourne, VIC Australia; 11 Sir Peter MacCallum Department of Oncology, The University of Melbourne, Melbourne, VIC 3000 Australia; 12 Florey Department of Neuroscience and Mental Health, The University of Melbourne, Melbourne, Australia

**Keywords:** Neratinib, Gut toxicity, Colonic injury, Ferroptosis

## Abstract

**Purpose:**

Neratinib, a small-molecule tyrosine kinase inhibitor (TKI) that irreversibly binds to human epidermal growth factor receptors 1, 2 and 4 (HER1/2/4), is an approved extended adjuvant therapy for patients with *HER2*-amplified or -overexpressed (*HER2*-positive) breast cancers. Patients receiving neratinib may experience mild-to-severe symptoms of gut toxicity including abdominal pain and diarrhoea. Despite being a highly prevalent complication in gut health, the biological processes underlying neratinib-induced gut injury, especially in the colon, remains unclear.

**Methods:**

Real-time quantitative polymerase chain reaction (RT-qPCR) and histology were integrated to study the effect of, and type of cell death induced by neratinib on colonic tissues collected from female Albino Wistar rats dosed with neratinib (50 mg/kg) daily for 28 days. Additionally, previously published bulk RNA-sequencing and CRISPR-screening datasets on human glioblastoma SF268 cell line and glioblastoma T895 xenograft, and mouse TBCP1 breast cancer cell line were leveraged to elucidate potential mechanisms of neratinib-induced cell death.

**Results:**

The severity of colonic epithelial injury, especially degeneration of surface lining colonocytes and infiltration of immune cells, was more pronounced in the distal colon than the proximal colon. Sequencing showed that apoptotic gene signature was enriched in neratinib-treated SF268 cells while ferroptotic gene signature was enriched in neratinib-treated TBCP1 cells and T895 xenograft. However, we found that ferroptosis, but less likely apoptosis, was a potential histopathological feature underlying colonic injury in rats treated with neratinib.

**Conclusion:**

Ferroptosis is a potential feature of neratinib-induced colonic injury and that targeting molecular machinery governing neratinib-induced ferroptosis may represent an attractive therapeutic approach to ameliorate symptoms of gut toxicity.

**Supplementary Information:**

The online version contains supplementary material available at 10.1007/s00280-024-04699-9.

## Introduction

Breast cancer is the most frequently diagnosed type of cancer in women worldwide [1]. Among all subtypes of breast cancer, the aggressive human epidermal growth factor 2-positive (*HER2*-positive) breast cancer, which is characterised by overexpressed or amplified HER2 receptor, accounts for approximately 20–30% of all annually diagnosed cases [2]. In the past century, patients diagnosed with *HER2*-amplified or -overexpressed (also referred as *HER2*-positive) breast cancer were frequently associated with the poorest prognosis and with the highest incidence of brain metastasis [3–7]. However, this is no longer the case following the relatively recent development of novel HER2-targeted therapies, including monoclonal antibody and small-molecule tyrosine kinase inhibitors (TKI), that more precisely target HER2-positive cells to inhibit the activation of downstream signalling while triggering cell death pathways [8].

Orally taken ATP-competitive TKIs, such as the pan-HER-TKI neratinib, have garnered considerable attention in recent years as an effective extended adjuvant HER2-targeted therapy for patients with *HER2*-positive breast cancers after receiving a combination of chemotherapy and monoclonal antibody trastuzumab [9]. Neratinib treatment significantly improves invasive disease-free survival rate while reducing long-term toxicity and complication rate. Mechanistically, neratinib forms a covalent bond with cysteine residues in the ATP-binding pocket of HER1 (also known as EGFR), HER2 and HER4 receptors to inhibit their downstream canonical Ras/ERK and Akt/mTOR pathways, suppressing cell proliferation, and triggering cell death [10, 11]. A previous study using *in vitro HER2*-positive breast cancer cell lines, which expressed wild-type oestrogen receptor, such as SKBR3, showed that neratinib treatment repressed the activity of nuclear factor-erythroid factor 2-related factor 2 (NRF2), a master regulator of antioxidant defence, to then promote oxidative-stress-dependent cell death [12]. Oxidative stress is a condition of sustained elevation of intracellular reactive oxygen species (ROS) that potentially leads to reversible and irreversible damage to biomolecules such as lipids, proteins, and nucleic acids [13]. ‘ROS’ is an umbrella term describing a collection of oxygen-derived radicals, such as singlet superoxide (O_2_^• −^) and hydroxyl radical (^•^OH), and species, such as hydrogen peroxide (H_2_O_2_) and lipid peroxide. Strikingly, a recent report from Nagpal and colleagues revealed that, rather than apoptosis as induced by other TKIs, such as lapatinib, neratinib promoted non-apoptotic cell death called ferroptosis in both human (SKBR3) and mouse (TBCP1) *HER2*-positive breast cancer cell lines [14]. Ferroptosis is a newly discovered type of cell death characterised by perturbed iron and redox homeostasis with excessive lipid peroxidation at phospholipid membrane [15]. Unless the rise of endogenous lipid peroxide is averted such as by the scavenging enzymatic activity of glutathione peroxidase 4 (GPX4), the accumulation of lethal membrane lipid peroxide results in the loss of membrane integrity and ultimately leads to cell death [16].

Despite therapeutic effectiveness against *HER2*-positive breast cancers, patients receiving neratinib can experience mild to severe symptoms of gut toxicity, such as abdominal pain and diarrhoea, which can lead to early dose reduction or treatment discontinuation [17, 18]. Interestingly, a recent randomised controlled trial conducted by Sonnichsen and colleagues reported that though serum levels of neratinib were higher in patients receiving 240 mg of neratinib once daily (QD) than those receiving 120 mg of neratinib twice daily (BID), patients in the QD group experienced less severe diarrhoea [18]. This suggested that diarrhoea is potentially a consequence of local exposure to neratinib rather than systemic exposure. In support, starting patients on a reduced dose of neratinib for the first two weeks is also associated with greatly reduced levels of diarrhoea and treatment discontinuations [19]. Furthermore, an escalating dose of neratinib resulted in more severe symptoms of gut toxicity observed in our preclinical rat models and other clinical trials [20, 21]. We therefore postulate that inhibition of locally expressed HER receptors in the intestinal epithelial cells which subsequently triggers epithelial cell death is likely a cause of gut toxicity. This is plausible due to the gut epithelium naturally expressing HER receptors and being strongly dependent on HER downstream signalling pathways for cell survival, differentiation, and proliferation [22–25]. As such, the disruption of HER signalling perturbs intestinal homeostasis leading to intestinal cell death and symptoms of gut toxicity.

In contemporary practice, patients are co-prescribed with loperamide to reduce the incidence of TKI-induced diarrhoea [26]. Loperamide acts by reducing gut motility and secretion by activating the µ-opioid receptors in the muscle wall, thus, reducing diarrhoea [27, 28]. However, data from the phase II CONTROL clinical trial suggest that escalating the dose of prophylactic loperamide alone, or in combination with other antidiarrhoeic medications only provided modest symptomatic relief following neratinib treatment [29, 30]. This modest relief may be because these prophylactic interventions do not adequately target the underlying cause of neratinib-induced gut toxicity, which remains poorly understood and may be dependent on intestinal epithelial cell death, especially in the colon.

In the present study, we sought to explore biological processes underlying neratinib-mediated colonic epithelial injury using clinically relevant in vivo healthy female Albino Wistar rats dosed with neratinib for 28 consecutive days [20]. We found that the colonic epithelium, especially the surface lining colonocytes, of the distal colon was more severely injured than that of the proximal colon. With further analyses from our rat study and the integration of published bulk RNA-sequencing and CRISPR-screening datasets [14, 31], we found that ferritinophagy-mediated ferroptosis, characterised by perturbed iron homeostasis and lipid peroxidation, rather than apoptosis, was the potential underlying histopathology of neratinib-induced cell death and colon injury.

## Materials and methods

### Rats

All in vivo experiments for healthy female rats dosed with either vehicle control (0.5% hydroxypropyl methyl cellulose; HPMC) or neratinib maleate (50 mg/kg) were previously approved by The University of Adelaide Animal Ethics Committee (M-2019-025) and were performed according to the Australian code for the care and use of animals for scientific purposes, 8th edition, 2013 (revised 2021).

All tissues were previously collected and described in Secombe et al. [32]. In brief, rats were treated by oral gavage (5 ml/kg) with either HPMC (0.5%) or neratinib (50 mg/kg) every day for 28 days consecutively (*n* = 6/group). Rats were culled by cardiac puncture while under 4% isoflurane anaesthesia. After colons were dissected and flushed with sterile saline, the length of colon was separated into proximal (the first 3 cm), mid and distal thirds (the last 3 cm). While the mid portion of the colon was snap frozen in liquid nitrogen before storage at -80^o^C, the proximal and distal portions were fixed in 10% neutral buffered formalin for 24 h. After fixation, tissues were processed and paraffin-embedded using standard techniques.

### Haematoxylin and eosin (H&E) staining and histopathological scoring

Tissue H&E staining was carried out to visualise intestinal pathology and score the injury associated with neratinib treatment. Archived paraffin-embedded blocks of proximal and distal colon collected from rats dosed with vehicle control or neratinib were cut into 5 μm sections on a rotary microtome (Leica, Germany). Tissue sections were first de-waxed in xylene (3 × 5 min) followed by rehydration in a series of graded ethanol concentrations (100% and 90%; 60 s each). Sections were then stained in Harris haematoxylin solution (1:10) for 5 min followed by eosin for 2 min. Sections were subsequently differentiated in acid-alcohol solution (1% concentrated hydrochloric acid in 70% ethanol; 2 quick dips) and Scott’s tap water for 2 min. Slides were dehydrated in a series of graded ethanol concentrations (90% and 100%; 30 s each) followed by clearing in xylene (3 × 5 min). Tissue slides were cover-slipped and mounted in Entellan New mounting media. All slides were scanned using Nanozoomer Digital Slide Scanner and viewed using the Nanozoomer Digital Pathology Software (NDP View v2.0, Histalim).

Criteria for assessing histopathological features in rat colon previously established by Howarth et al. (1996) were used for histopathological scoring on H&E slides [33]. These criteria included disruption of surface colonocytes, crypt loss or disruption, disruption of crypt cells, infiltration of immune cells, dilation of lymphatics and capillaries, and oedema. Each criterion was scored from 0 to 2, whereby 0 indicated no apparent morphological changes, 1 indicated mild damage, and 2 indicated severe damage.

### Immunohistochemical (IHC) staining

IHC staining was carried out to visualise (1) the changes of markers of surface colonocytes (CA1) and proliferative cells (Ki67) in support for histopathological assessment; and (2) the changes of key markers of ferroptosis, namely ferritin heavy chain 1 (FTH1) (Table 1). Proximal and distal colon blocks collected from vehicle-control- and neratinib-treated rats were cut into 4 μm sections on a rotatory microtome (Leica, Germany). Slides were then processed as follows: de-waxed in xylene/histolene (3 × 5 min) followed by rehydration in a series of graded ethanol concentrations (100%, 90% and 70%; 1 min each). Thereafter, slides underwent heat-mediated antigen retrieval in Tris/EDTA (pH 9.0) buffer depending on the primary antibody used as described in Table 1. After pre-heating antigen retrieval buffer to 65^o^C, slides were immersed and heated to 97^o^C for 20 min. Temperature was left to return to 65^o^C before slides were transferred to the Dako Autostainer instrument. Next, endogenous peroxidase was blocked using Dako REAL peroxidase blocking solution for 10 min followed by blocking non-specific protein using DAKO protein block solution for 30 min. Primary antibody diluted in EnVision™ FLEX Antibody Diluent was applied on tissue sections and incubated for 60 min. Primary antibody was then detected using either secondary-HRP labelled mouse or rabbit antibody detection system (Dako EnVision^+^ System-HRP; 30 min) followed by the addition of 3,3ʹ-diaminobenzidine (DAB) chromogen (10 min) was added for visualization. Sections were counter-stained with Harris haematoxylin solution (1:10) for 5.0 min followed by differentiating in acid-alcohol solution (1% hydrochloric acid in 70% ethanol; 2 quick dips) and Scott’s tap water for 2 min. Slides were dehydrated in 70%, 90% and 100% ethanol (30 s each) before clearing in xylene (3 × 5 min). Slides were cover-slipped and mounted in Entellan New mounting media. A negative control omitting the primary antibody, as well as a positive control showing the normal pattern of expression of the protein of interest, were run with each batch of IHC staining. All slides were scanned using Nanozoomer Digital Slide Scanner and viewed using the Nanozoomer Digital Pathology Software (NDP View v2.0, Histalim). Caspase-3-positive cells in 10 randomly selected intact crypts per animal were counted and data were expressed as the average of positively stained cells per crypt. Ki67-positive cells from 10 randomly selected intact demi (or half) crypts per animal were counted. Data were expressed as the percentage of the average of positively stained cells per crypt using the following equation:

**Table 1:** A list of antibodies, and their recommended antigen retrieval buffer and dilution used for IHC staining

Antibodies	Source	Identifier	Antigen retrieval buffer	Dilution
Rabbit polyclonal anti-Caspase-3 antibody	Abcam	ab4051	Tris/EDTA buffer	1:100
Rabbit recombinant anti-Ferritin (FTH1) antibody	Abcam	ab287968	Tris/EDTA buffer	1:2,000
Rabbit recombinant Anti-Ki67 antibody [SP6]	Abcam	ab16667	Tris/EDTA buffer	1:100
Rabbit recombinant Anti-Carbonic Anhydrase 1 (CA1)	Abcam	ab267475	Tris/EDTA buffer	1:2,000

$$\begin{aligned}\%{Ki67}^{+}\:cells\:per\:crypt&=\frac{\begin{array}{l}{\left(Number\:of\:{Ki67}^{+}\right.}\\{\left.\:cells\:per\:demi\:crypt\right)\;\times\:2}\end{array}}{Total\:number\:of\:cells\:per\:crypt}\\ &\quad\times\:100\:\left(\%\right)\end{aligned}$$

### Real-time quantitative PCR (RT-qPCR)

RT-qPCR was utilised to quantify the expression of key markers of ferroptosis in rat colons (Table 2). All primers were purchased from Integrated DNA Technologies, Inc. (USA). Total ribonucleic acid (RNA) extraction and purification of rat mid colons were carried out using NucleoSpin RNA extraction kit following the manufacturer’s instructions. The concentration (ng/µL) and purity of extracted RNA was determined using Synergy™ Mx reader (BioTek, USA). 1 µg of RNA was reverse transcribed into cDNA using iScript™ cDNA Synthesis Kit using the following steps – priming at 25^o^C for 5 min, reverse transcription (RT) at 46^o^C for 20 min, and RT deactivation at 95^o^C for 1 min. RT-qPCR was subsequently carried out using the SYBR-Green QuantiTect system on the Rotor-Gene qPCR cycler (Qiagen). Amplification mixture of RT-qPCR contain 1 µL of cDNA (100 ng), 5 µL of SYBR green dye, 3 µL of nuclease-free water, and 0.5 µL of each forward and reverse primers (50 pmol). All samples were run in triplicate. Raw data was plotted as cycle (x-axis) against normalised fluorescent signal (y-axis). Cycling threshold (Ct) value was calculated by Rotor Gene 6 analysis software and used for relative quantification reported in fold difference. Then, the fold difference in RNA expression between groups was determined using ΔΔCt method established by Schmittgen and Livak [34]. All genes were normalised to Ubiquitin C (*Ubc*) housekeeping gene. The melting curve analysis was additionally performed to examine the presence of primer-dimers and specificity of PCR product.

**Table 2:** List of primers used for RT-qPCR experiments

Target mRNA	NCBI reference sequence	Forward primer (5’-3’)	Reverse primer (5’-3’)
Ubiquitin C (Ubc) - *Rattus norvegicus*	NM_017314.1 (Ref. [62])	TCGTACCTTTCTCACCACAGTATCTAG	GAAAACTAAGACACCTCCCCATCA
Transferrin receptor (Tfrc) - *Rattus norvegicus*	NM_022712.1	CGGCTACCTGGGCTATTGTA	TTCTGACTTGTCCGCCTCTT
Ferritin heavy chain 1 (Fth1) - *Rattus norvegicus*	NM_012848.2	ATGATGTGGCCCTGAAGAAC	CACACTCCATTGCATTCAGC
Arachidonate 15-lipoxygenase (Alox15) - *Rattus norvegicus*	NM_031010.2	CTTCCTTCTGGATGGGATCA	ATGGCTATGGGCAAGAGTTG
Acyl-CoA synthetase long-chain family member 4 (Acsl4) - *Rattus norvegicus*	NM_053623.1	TTGAAGTGAACTGCCGAGTG	CACAGAAAATGGCAATGGTG
Glutathione peroxidase 4 (Gpx4) - *Rattus norvegicus*	NM_001368043.1	TACGAATCCTGGCCTTCCCT	CCCTTGGGCTGGACTTTCAT

### RNA-sequencing analysis

RNA-sequencing analysis on previously published datasets was carried out to examine which biological pathways were significantly enriched following neratinib treatment. Processed count tables for protein encoded genes from neratinib-treated mouse TBCP-1 breast cancer and human glioblastoma SF268 and TS895 cell lines were kindly provided by Dr Normand Pouliot (Olivia Newton-John Cancer Research Institute) and Dr Colin Tang (Weill Cornell Medicine) [14, 31]. Differential gene expression between neratinib and vehicle control groups was performed using DESeq2 (v1.38.3) statistical package in R [35]. Unless otherwise stated, a master list of differentially expressed (DE) genes was generated when the P-adjusted value less than 0.05. Subsequently, a list of significantly upregulated genes with log_2_FoldChange values above 0.1 was generated from the master DE gene list with P-adjusted values or false discovery rate (FDR) below 0.05. Subsequently, this gene list was inserted into the online database for annotation, visualisation, and integrated discovery (DAVID) bioinformatic resource website for gene ontology (GO) terms and KEGG pathway analyses as previously described [36–38]. The computational algorithm used to determine statistical significance of individual pathways was detailed in the original papers [36, 39]. Here, GO terms and KEGG pathways were deemed as significantly enriched with P values below 0.05.

### CRISPR-screening analysis

As previously described in Tang et al. [31], a pool CRISPR screen was performed on neratinib-treated SF268 human glioblastoma cell line to identify genes contributing to neratinib sensitivity. A count table of differentially expressed single-guided RNA and an output file of DrugZ were kindly provided by Dr Colin Tang from Weill Cornell Medicine. Using R script publicly available generated by Dr Colin Tang, drugZ rank plot was generated for normalised Z score against sgRNA rank ^95^. The positive or negative normalised Z score of a gene with FDR below 0.05 confers with either neratinib sensitivity or resistance, respectively.

### Data analysis and statistics

Descriptions for sample size and statistical tests are detailed in figure legends. Unless otherwise specified, all statistical analyses were carried out using unpaired Student’s t-test in GraphPad Prism 9 (version 9.2.0; USA). Statistical values were reported in mean ± standard error of the mean (mean ± SEM). Statistical significance, which was reported as exact P values, was considered as followed – for all histological assessment, RT-qPCR, gene ontology and KEGG pathway analyses, P values below 0.05 were deemed significant while for differential gene expression and Drug-Z analyses, P-adjusted values, or false discovery rate (FDR) below 0.05 were deemed significant.

### Data availability

The data generated in this study are available within the article and its supplementary data files, with further raw data available upon request. Further information and requests for resources should be directed to and will be fulfilled by the corresponding authors, Joanne M. Bowen (joanne.bowen@adelaide.edu.au) and Triet PM. Nguyen(phuminhtrietthomas.nguyen@unimelb.edu.au).

## Results

### Neratinib-induced injury is spatially located in the rat colon

**Fig. 1:**
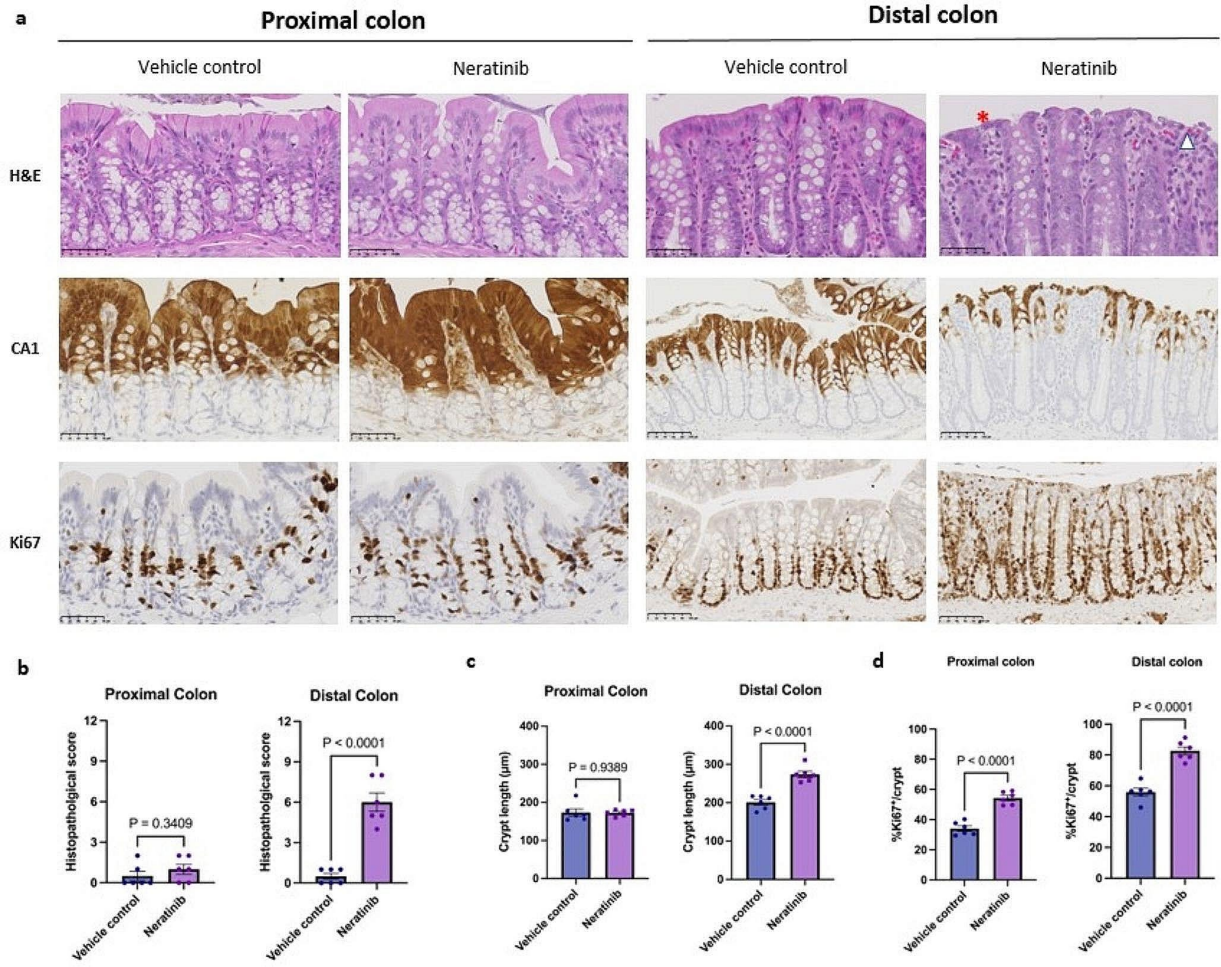
Histopathological features of neratinib-induced injury in proximal and distal colon. **a**, Representative images of H&E panels of proximal and distal colon proximal and distal colon treated with either vehicle control or neratinib at 20X and 40X magnification. In the distal colon of neratinib-treated rat, (*) indicates denuded mucosal lining is covered by attenuated, surviving enterocytes in an attempt to repair the injured surface lining colonocytes and **(Δ)** indicates a marked lymphocytic infiltrate in the lamina propria. Scale bar, 100 mm. Representative images of IHC (CA1 and Ki67) panels of proximal and distal colon of rats treated with either vehicle control or neratinib. Scale bar, 50 mm (proximal colon) and 100 mm (distal colon) **b**, Quantification of histopathological scoring. **c**, Quantification of crypt length. **d**, Quantification of the percentage of Ki67-postive cells per crypt. For **b**,** c**, and **d**, *n* = 6 rats in each treatment group. Unpaired Student’s t-test was used for statistical analysis, where P values below 0.05 were deemed statistical significance. The centre line represents the mean, and the error bar represents s.e.m

As previously reported from our group, all rats dosed with neratinib experienced common signs of gut toxicity including weight loss and moderate diarrhoea [32]. To determine the spatial effect of neratinib on the rat colon, we performed histopathological assessment using haematoxylin and eosin (H&E) and immunohistochemical (IHC) staining on proximal and distal colon. Overall, we observed that following neratinib treatment, the most severe morphological damage was found in the distal colon as reflected by a significantly higher histopathological score and increased crypt length (Fig. 1a, b, and c). In sharp contrast, despite a significant increase in proliferative Ki67-positive cells, the proximal colon of neratinib-treated rats only displayed a few, small, focal areas of mild mucosal damage without apparent sign of injury in the surface lining colonocytes expressing carbonic anhydrase 1 (CA1) (Fig. 1a-d).

In the distal colon, degeneration of surface lining CA1^+^ colonocytes with occasional exfoliation of epithelial cells into the lumen leaving a residual denuded surface was observed (Fig. 1a). Migration of flattened, attenuated enterocytes across the denuded mucosal surface was also found which suggested that surviving epithelial cells attempted to cover the surface defects to maintain the integrity of the epithelial lining (Fig. 1a). A significant increase in the proliferative crypt compartment containing Ki67-positive cells further suggested that a reparative mechanism was in place to regenerate the injured surface epithelium (Fig. 1d). A markedly increased inflammatory infiltrate, which primarily comprised of lymphocytes, but also a few granular leukocytes, mostly eosinophils, were observed in the lamina propria layer of the distal colon following the exposure to neratinib compared to vehicle control (Fig. 1a). Together, these histopathological findings appeared to consistent with features of microscopic colitis, specifically lymphocytic colitis [40]. Our current histopathological data collectively substantiated our observations that the injury in the distal colon and its surface lining colonocytes appeared to be more severe than in the proximal colon on day 28 of neratinib treatment.

### Enriched gene signatures for apoptosis or ferroptosis may be cell-type specific following neratinib treatment

**Fig. 2:**
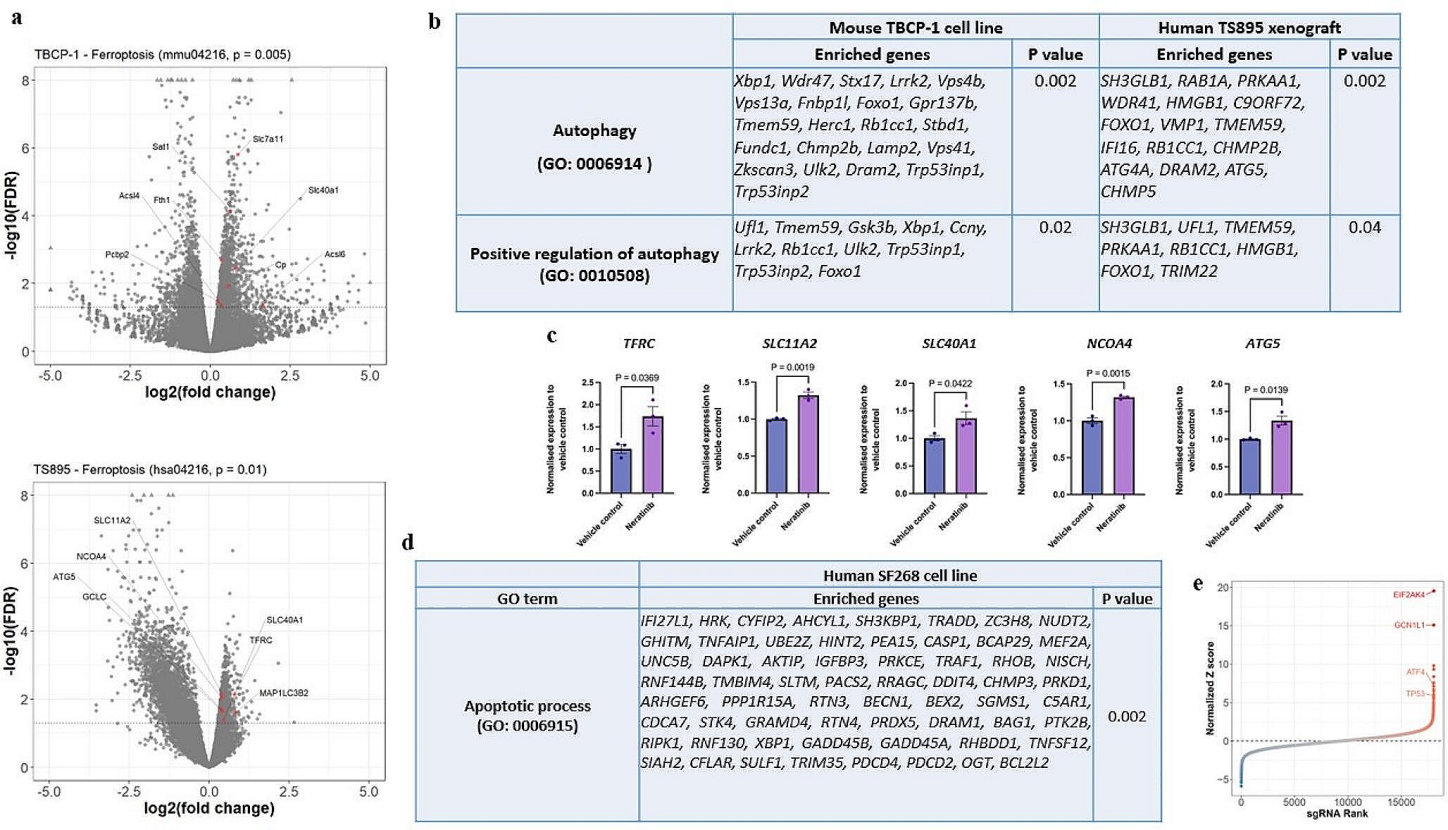
Whether apoptosis or ferroptosis is induced following neratinib treatment may be cell-type specific. **a**, Volcano plot for upregulated key markers of ferroptosis analysed from published bulk RNA-sequencing data of TBCP-1 cell line and TS895 xenograft following 24 h and 3 h of neratinib treatment, respectively. **b**, A table of enriched gene sets in autophagy and positive regulation of autophagy following neratinib treatment in TBCP-1 cell line (24 h) and TS895 xenograft (3 h). **c**, Differential gene expression of selected markers for iron transport and ferritinophagy in ferroptotic pathway from KEGG pathway analysis in TS895 xenograft. Unpaired Student’s t-test was used for statistical analysis, where P values below 0.05 were deemed statistical significance. The centre line represents the mean, and the error bar represents s.e.m. **d**, A table of enriched gene sets in apoptotic process following neratinib treatment in SF268 cell line (72 h). **e**, Gene candidates corresponding to neratinib resistant phenotype identified from SF268 CRISPR screen experiment

To gain insight into the biological processes underlying neratinib-induced cell death, we performed comprehensive analyses on differential gene expression, KEGG pathway and GO on previously published RNA-sequencing datasets [14, 31]. These datasets included TBCP-1 cells, a mouse *HER2*-overexpressing breast cancer cell line capable of metastasising to the brain, treated with neratinib for 24 h, and intracranial human *EGFR/HER1*-mutant TS895 glioblastoma xenograft treated with neratinib for 3 h [14, 31]. We decided to utilise these datasets for this analysis because of the absence of -omics studies in neratinib-treated healthy colonocytes or at least in colorectal cancer cell lines in vitro. Our KEGG pathway analysis revealed that ferroptotic cell death, which is characterised by perturbed iron homeostasis and lipid peroxidation [41], was significantly enriched in both model systems (Fig. 2a). As a previous study by Nagpal and colleagues also confirmed that ferroptosis was induced by neratinib in *HER2*-postive human (SKBR3) and mouse (TBCP1) breast cancer cell lines [14], we hypothesised that neratinib-induced ferroptosis might extend beyond the context of breast cancer.

Gene Ontology (GO) analyses for enriched biological processes further uncovered gene signatures involved in autophagy and positive regulation of autophagy among all biological processes in the list of significantly upregulated genes in both neratinib-treated TBCP1 and TS895 cells (Fig. 2b and Suppl. Table 1). Autophagy is a process mediated by the lysosomal pathway [42] and was previously related to the induction of ferroptosis, especially in the form of ferritinophagy [43, 44]. Ferritinophagy is a process of breaking down ferritin, an intracellular iron storage protein, to release reactive ferrous ion that then accelerates lipid peroxidation [45]. We, therefore, asked if ferritinophagy-mediated ferroptosis was a likely initiating event of neratinib-induced ferroptosis. Indeed, among all differentially expressed genes contributing to ferroptosis that were identified from the KEGG pathway analysis, only genes involved in iron transport, such as transferrin receptor (*TFRC*), divalent metal transporter 1 (*SLC11A2*) and ferroportin-1 (*SLC40A1*), and ferritinophagy, such as nuclear receptor coactivator 4 (*NCOA4*) and autophagy related 5 (*ATG5*), were significantly upregulated in TS895 cells following a 3-hour neratinib treatment (Fig. 2c and Suppl. Table 1). On the other hand, genes involved in both iron metabolism, such as ferritin heavy chain 1 (*Fth1*) and *Slc40a1*, and lipid peroxidation, such as acyl-CoA synthetase long chain family member 4 and 6 (*Acsl4/6*), were significantly upregulated in the TBCP-1 cell line following a 24-hour neratinib treatment (Fig. 2a and Suppl. Table 1). Together with a significant increase in iron uptake previously observed in neratinib-treated TBCP-1 cells [14], our bulk RNA-sequencing analyses collectively suggested that *HER1*-mutant TS895 and *HER2*-positive TBCP1 cancer cells potentially underwent ferroptotic cell death, which appeared to arise from perturbed iron homeostasis through ferritinophagy, following neratinib treatment.

Intriguingly, our re-analyses of published bulk RNA-sequencing and CRISPR-screening datasets of neratinib-treated human SF268 glioblastoma cell line suggested that p53-dependent apoptosis, but not ferroptosis, was induced following 72 h of neratinib treatment (Fig. 2d and e). This then prompted us to propose that the type of cell death induced by neratinib, i.e., ferroptosis or apoptosis, may depend on cell type and duration of neratinib treatment. Together with the histopathological assessment from the colon of rats dosed with neratinib, these findings motivated us to explore the biological process, especially the type of cell death, underlying neratinib-induced colon injury.

### Ferroptotic cell death is a potential feature underlying neratinib-induced colonic epithelial injury

**Fig. 3:**
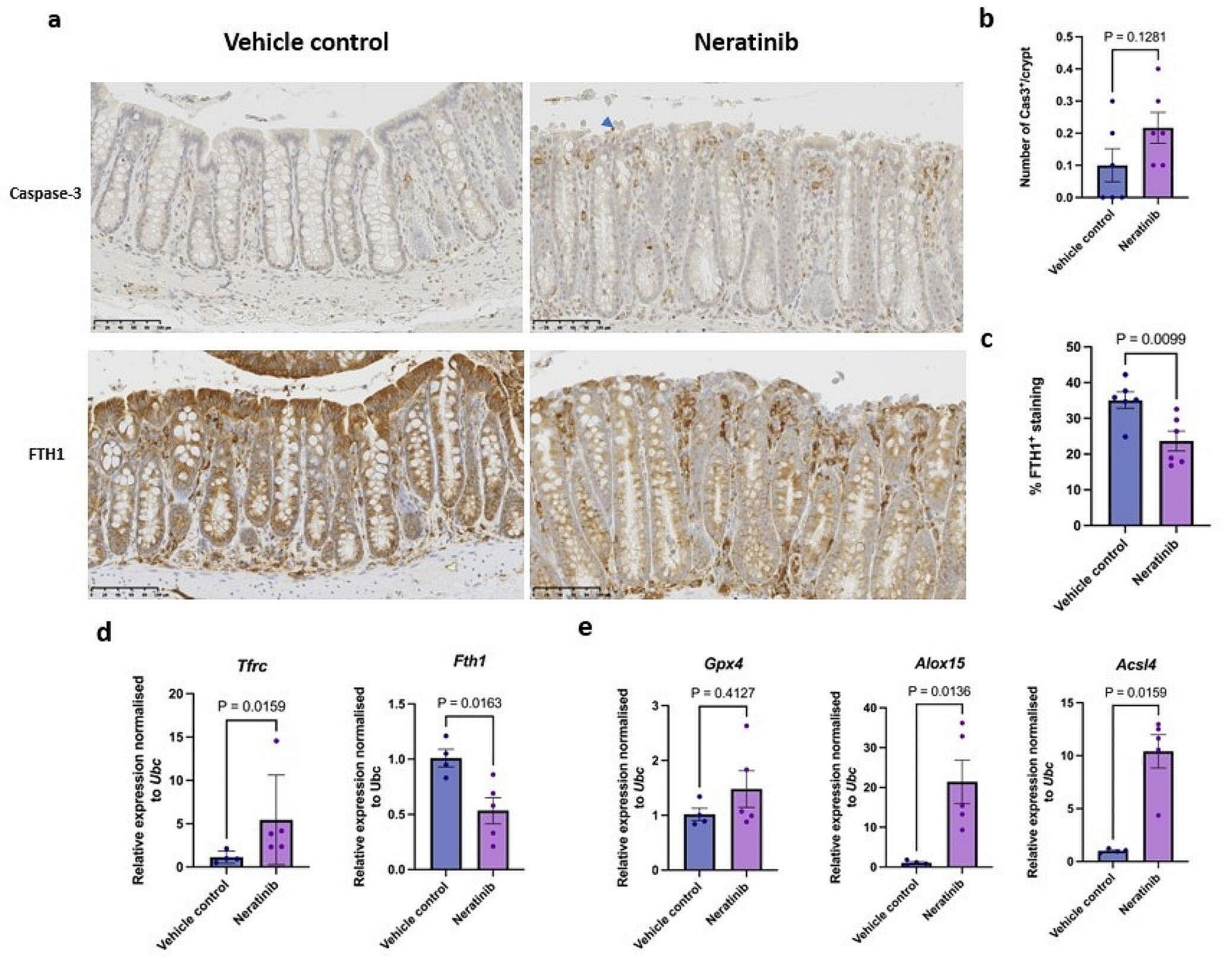
Ferroptosis is a potential underlying histopathological feature of neratinib-induced injury in the distal colon. **a**, Representative images of IHC-stained (Caspase-3 and FTH1) distal colon treated with either vehicle HPMC or neratinib. Arrowhead indicates Caspase-3-positive cell. Scale bar, 100 mm. **b**, The quantification of positive Caspase-3 cells per crypt. **c**, The gene expression levels of markers for iron metabolism (*Tfrc* and *Fth1*). **d**, The gene expression levels of markers for lipid peroxidation, namely *Gpx4*, *Alox15*, and *Acsl4*. For **b**,** c**, and **d**, *n* = 4 rats in vehicle-treated group, and *n* = 5 in neratinib-treated group. The gene expression levels were determined by RT-qPCR and were shown relative to *Ubc* housekeeping gene. Except non-parametric Mann-Whitney test was used for *Tfrc*, *Gpx4*, and *Acsl4*, unpaired Student’s t-test was used for statistical analysis, where P values below 0.05 were considered significant. The centre line represents the mean, and the error bar represents s.e.m

As our histopathological findings revealed that neratinib caused degeneration of surface epithelial cells with strong inflammatory infiltrates in the distal colon (Fig. 3a) and our bulk RNA-sequencing analysis suggested neratinib could trigger either apoptosis or ferroptosis, this prompted us to question which type of cell death was likely induced by neratinib in the rat colon. To determine the presence of apoptosis, we performed IHC staining for Caspase-3 in the rat distal colon. We did not detect a significant change in Caspase-3-positive cells between the vehicle- and neratinib-treated rats (Fig. 3a and b). This suggested that apoptotic cell death may not be a prominent form of cell death in the rat colon following neratinib treatment, or at least at the timepoint investigated, which was 24 h after the final dose following 28 days of neratinib treatment.

We next aimed to determine the presence of ferroptosis by measuring key markers of iron homeostasis and lipid peroxidation. Through IHC staining, we observed a significant reduction of endogenous ferritin heavy chain 1 (FTH1) in the distal colon of rats treated with neratinib (Fig. 3a and c). A significant upregulation of expression of iron absorption gene, *Tfrc*, and downregulation of iron storage gene, *Fth1*, measured by RT-qPCR further suggested that neratinib perturbed the pool of intracellular iron storage leading to an increase in cytosolic reactive ferrous (Fe^2+^) ion in the rat colon (Fig. 3d). Although neratinib did not alter gene expression of *Gpx4*, which encodes a lipid peroxide scavenging enzyme contributing to ferroptotic resistance [16, 46], we found a significant upregulation of gene expression of *Acsl4*, which esterifies polyunsaturated fatty acids (PUFA) making them competent for ferroptosis, and arachidonate 15-lipoxygenase (*Alox15*), which directly causes lipid peroxidation [47, 48] (Fig. 3e). Collectively, the current findings support the notion that ferritinophagy-mediated ferroptosis, but not apoptosis, is the potential histopathological feature underlying neratinib-induced colonic epithelial injury.

## Discussion

In the present study, we used colonic tissues collected from healthy female Albino Wistar rats orally dosed with neratinib (50 mg/kg) daily for 28 consecutive days as an in vivo model for studying neratinib-induced colonic epithelial injury [20, 32]. Overall, our study reveals that the injury is spatially located predominantly in the distal colon. In particular, the degeneration and morphological changes of CA1-positive colonocytes lining the surface epithelium are more pronounced in the distal colon than in the proximal colon. We further suggest that ferroptosis is a potential histopathological feature underlying neratinib-induced colonic epithelial injury.

The spatial difference in the severity of injury observed in the proximal and distal colon could be explained on the basis of the molecular regionalisation of the colon. Using spatial transcriptomics, a recent study led by Villablanca’s team revealed that the difference in the morphology of the proximal and distal colon was governed by molecular regionalisation of gene expression [49]. They further showed that the severity of dextran sulphate sodium (DSS)-induced injury varying between different regions of the colon was likely due to this pattern. As the morphology of the proximal and distal colon of the rat are distinct as observed in representative H&E-stained sections (Fig. 1a), we suggest that similar to the mouse colon, there might exist a pattern of molecular regionalization that potentially implicated the distal colon as the major site of injury following neratinib treatment. For example, factors that may contribute to the severity of the injury following neratinib treatment could include the different distribution of HER receptors and upregulation of key inflammatory pathways such as JAK-STAT and TNFα pathways between the proximal and distal colon.

Alternatively, since all rats were culled on day 28 of neratinib treatment and a significant increase in proliferative Ki67-positive cells in both proximal and distal colon of rats treated with neratinib was apparent (Fig. 1a and d), we cannot exclude the possibility that mucosal damage may equally occur in both proximal and distal colon and that the proximal colon may possess an intrinsic adaptive mechanism that leads to a more rapid healing rate. This was reflected by a significant increase in Ki67-positive cells with a less severe injury phenotypes in the proximal colon on day 28 of neratinib treatment. Though the current study does not provide a precise explanation for why the injury in the distal colon appears to be more severe than in the proximal colon, it sheds light on the potential consequence of the pathology following neratinib treatment.

Further investigations in rat colon tissues and the integration of published bulk RNA-sequencing analyses revealed that ferritinophagy-mediated ferroptosis may be the underlying feature of not only colon injury, especially in the surface lining colonocytes, but also cell death in the HER2-positive TBCP-1 breast cancer cell line and HER1-mutant TS895 glioblastoma xenografts. However, there are several limitations associated with the current rat models that prevent a conclusive determination that ferroptosis is the causality of neratinib-induced colon injury. Firstly, though the upregulation of gene expression of *Acsl4* and *Alox15* is usually associated with lipid peroxidation, confirming the presence of lipid peroxide through IHC staining using HNEJ-1 or anti-malondialdehyde (MDA) antibodies are required to further substantiate the findings of ferroptosis [41, 50]. Secondly, a change in *Gpx4* gene expression was not observed in the neratinib-treated group. Previous studies found that transcriptional regulation of *Gpx4* did not seem to correlate with ferroptosis vulnerability, but rather mRNA translation and post-translational regulation of *Gpx4*, which leads to insufficient level or dysfunctional GPX4 enzymatic activity, is implicated in susceptibility to ferroptosis [16, 51–53]. Indeed, in the TBCP-1 breast cancer cell line, though neratinib did not alter GPX4 protein level, neratinib downregulated SLC3A2 expression and cysteine import. As a result, intracellular level of glutathione was depleted that potentially leads to the loss of scavenging enzymatic activity of GPX4 [54]. Altogether, we suggest that the enzymatic function of GPX4 might have been impeded in rat colon following neratinib treatment and that further research should be carried out to characterise the role of GPX4 in mediating neratinib-induced ferroptosis in the context of gut toxicity.

Finally, the degeneration changes in the morphology of surface lining CA1-positive colonocytes and changes in the expression of several markers of ferroptosis following a single endpoint of neratinib treatment are not sufficient to claim that ferroptosis is induced in colonocytes as a direct consequence of neratinib treatment. First, the degeneration of surface lining epithelial cells could be due to the improper differentiation of stem/progenitor cells that prevents CA1-positive colonocytes from adopting mature phenotypes, such as expressing carcinoembryonic antigen-related cell adhesion molecule 7 (CEACAM7) [55]. Second, following neratinib treatment, there was a dramatic increase in the infiltration of lymphocytes in the lamina propria and intraepithelial space of the distal colon that suggested a diagnosis of lymphocytic colitis. A common feature of lymphocytic colitis is the infiltration of CD8^+^ lymphocytes. Active immune cells, such as CD8^+^ lymphocytes, are able to stimulate ferroptotic cell death. Two recent studies from Wang et al. [56] and Liao et al. [57] reported that ferroptosis could be induced by active CD8^+^ T cells. Mechanistically, following the release of interferon gamma (IFNγ) from active CD8^+^ T cells and interaction with cognate receptors on melanoma and colon cancer cells, the downstream signalling of IFNγ robustly induced ferroptotic cell death by stimulating the uptake of arachidonic acid and upregulation of ACSL4 while suppressing the expression of SLC7A11, which encodes for system X_c_^−^ essential for cysteine uptake [56, 57]. As such, based on the current data collected from a single endpoint on day 28 of neratinib treatment, it is possible that ferroptosis is a consequence of active immune cells, such as CD8^+^ T cells, leading to epithelial cell death following neratinib treatment. Collectively, it is essential for future studies to determine whether (i) mature CEACAM7-positive colonocytes are the major target of neratinib and (ii) the death of surface lining colonocytes is a direct consequence of neratinib treatment or active CD8^+^ T cells.

To address the current gap in our in vivo rat model analysis and to further dissect the underlying mechanism of neratinib-induced colonic cell death, we provide several suggestions to direct future studies. First, several earlier timepoints of neratinib-treated rats is required to (i) assess the injury in both proximal and distal colons and (ii) to confirm whether the population of surface-lining CEACAM7-positive colonocytes is affected following neratinib treatment. Similar to a previous study by Chen and colleagues [50] where the presence of ferroptotic tissue was observed in a mouse model of DSS-induced colitis, to ascertain if ferroptosis is induced and dependent to ferritinophagy in rat colon, rats could be co-treated with neratinib and liproxstatin-1, a potent spiroquinoxalinamine radical trap of lipid peroxide [51], and deferoxamine (DFO) or deferiprone, an iron chelator scavenging reactive ferrous iron [15, 58]. If liprostatin-1 and DFO/deferiprone can substantially mitigate colon injury and signs of gut toxicity in rats treated with neratinib, then ferroptosis is confirmed as the underlying feature of neratinib-induced colon injury.

Second, future studies can potentially utilise in vitro three-dimensional colonic organoids, which can precisely recapitulate the complex heterogeneity of the colonic epithelium, to assess the direct effect of neratinib treatment on colonic epithelial cells avoiding the contribution of the immune system [59–61]. Critically, the use of undifferentiated stem and proliferative cells, and differentiated immature and mature colonocytes, organoids derived from both humans and mice are recommended to investigate the effects of neratinib inhibition on the differentiation potential of undifferentiated organoids and cell death of differentiated colonocytes.

In summary, the findings from this study suggest that ferritinophagy-mediated ferroptosis is a potential mechanism of cell death driving neratinib-induced colonic mucosal injury. We hope that our current findings will inspire future research to pursue a thorough mechanistic elucidation of how neratinib induces ferroptosis. An insight into this biological phenomenon may serve as an exciting new platform for future supportive therapies and drug discovery to mitigate gut toxicity while enhancing the efficacy of similar or emerging anti-cancer therapeutics.

## Electronic supplementary material

Below is the link to the electronic supplementary material.

Supplementary Material 1

## Data Availability

No datasets were generated or analysed during the current study.
